# Transport of the Influenza Virus Genome from Nucleus to Nucleus

**DOI:** 10.3390/v5102424

**Published:** 2013-10-02

**Authors:** Edward C. Hutchinson, Ervin Fodor

**Affiliations:** Sir William Dunn School of Pathology, University of Oxford, South Parks Road, Oxford OX1 3RE, UK; E-Mail: ervin.fodor@path.ox.ac.uk

**Keywords:** influenza virus, nuclear export, intracellular transport, genome packaging, viral entry, nuclear import, NEP, Rab11

## Abstract

The segmented genome of an influenza virus is encapsidated into ribonucleoprotein complexes (RNPs). Unusually among RNA viruses, influenza viruses replicate in the nucleus of an infected cell, and their RNPs must therefore recruit host factors to ensure transport across a number of cellular compartments during the course of an infection. Recent studies have shed new light on many of these processes, including the regulation of nuclear export, genome packaging, mechanisms of virion assembly and viral entry and, in particular, the identification of Rab11 on recycling endosomes as a key mediator of RNP transport and genome assembly. This review uses these recent gains in understanding to describe in detail the journey of an influenza A virus RNP from its synthesis in the nucleus through to its entry into the nucleus of a new host cell.

## 1. Introduction

**Figure 1:**
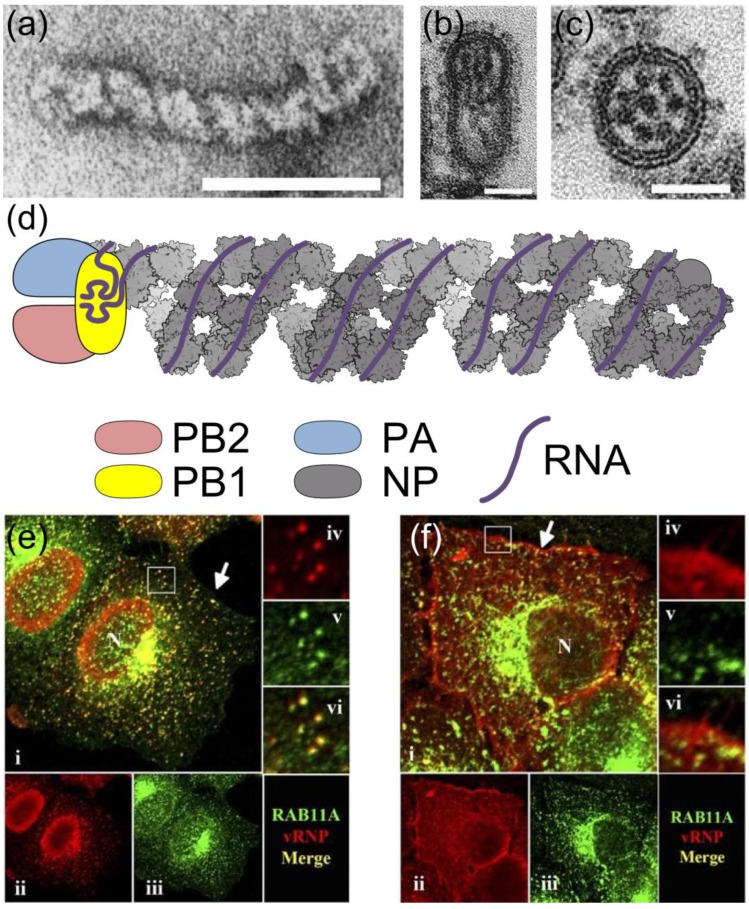
The influenza A virus ribonucleoprotein (RNP). The negative-sense RNA of the influenza A virus genome is divided into eight segments, each of which is encapsidated into an RNP. Electron micrographs showing (**a**) a negatively-stained RNP and (**b**) longitudinal and (**c**) transverse views of budding virions; scale bars are 50 nm. In (**b**) and (**c**) a complex of eight RNPs is visible as dark rods or dots. (**d**) Diagram of RNP structure. The NP backbone is based on reference [9] (PDB 4BBL, illustrated using the Python Molecular Viewer [14]); the polymerase and viral RNA, including the terminal promoter structure bound by PB1, are shown schematically. (**e**, **f**) Localisation of RNPs in an infected cell. Infected cells were fixed at 7 h (**e**) or 11 h (**f**) post-infection, and labelled with antibodies against RNPs (red, **ii** and **iv**) and the Rab11 isoform Rab11A (green, **iii** and **v**). The selected regions are enlarged 3× in frames **iv**, **v** and **vi**. As the infection progresses RNPs are exported from the nucleus (N) and associate with Rab11 in a perinuclear region for transport across the cytoplasm, then dissociate from Rab11 at the plasma membrane (arrow). Images in (**a**) reprinted from [15] with permission from Elsevier; in (**b**, **c**) adapted by permission from Macmillan Publishers Ltd: Nature [16], copyright (2006) and in (**e**, **f**) reproduced from [17] with permission from American Society for Microbiology.

**Figure 2:**
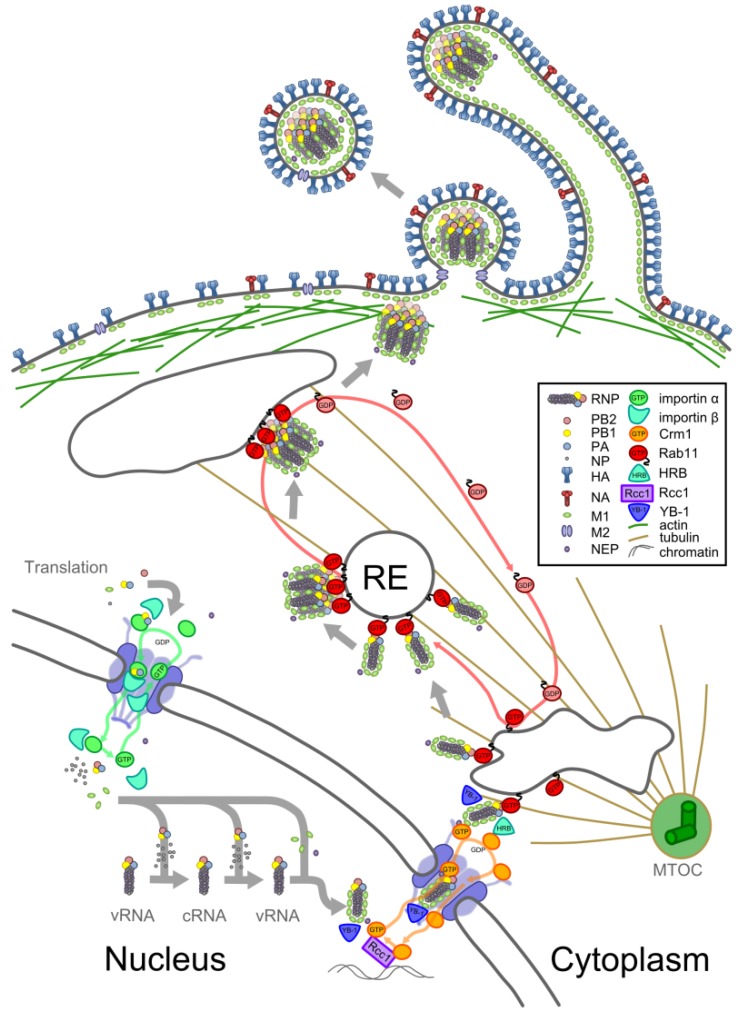
Transport of an RNP from synthesis to packaging. Schematic showing the synthesis and transport of an RNP within an infected cell. Viral proteins are translated in the cytoplasm and imported into the nucleus (**bottom left**). Here, some encapsidate replicating viral RNA, forming RNPs, and others bind to these newly formed RNPs. Assembled RNPs are exported from the nucleus and attach to recycling endosomes (RE) at the nuclear periphery. On REs they associate to form complexes and are transported away from the microtubule organising centre (MTOC) to the cell surface (**top**). At the cell surface, complexes of RNPs are packaged with other viral proteins into virions, which can be spherical or filamentous in morphology. See text for further details.

**Figure 3:**
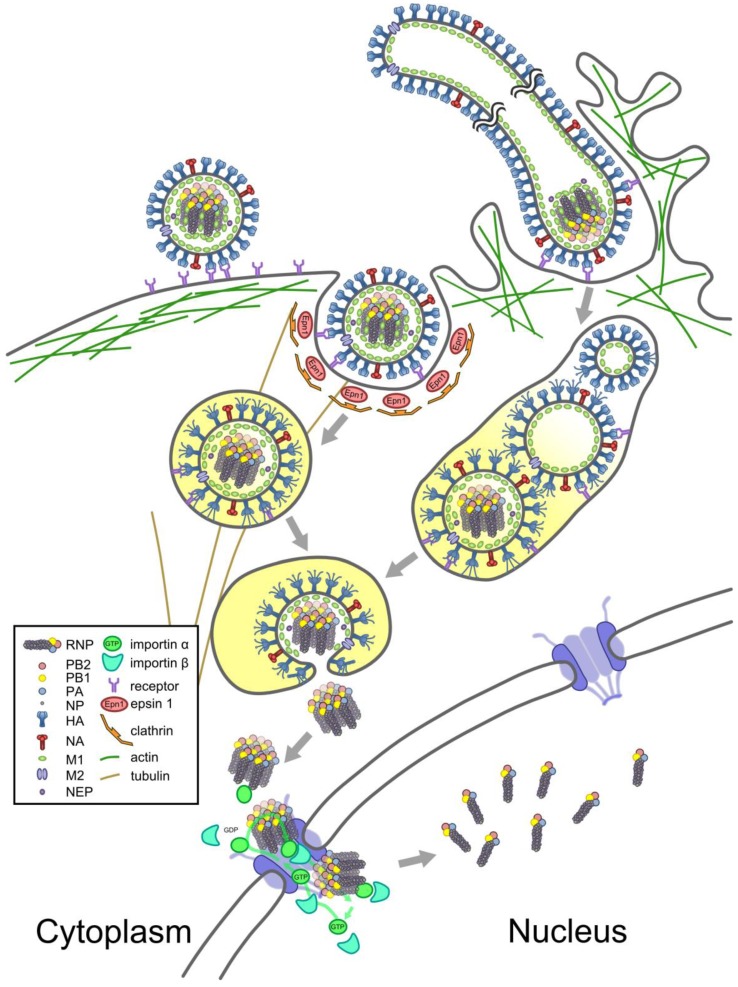
Transport of an RNP from virion to nucleus. Schematic showing viral entry and the passage of RNPs to the nucleus. Spherical (**left**) and filamentous (**right**) virions bind to sialic-acid bearing receptors on the cells surface and are imported by clathrin-mediated endocytosis (**left**) or macropinocytosis (**right**). The increasing acidity of their environment (indicated by yellow shading) results in conformational changes in M2, causing filaments to fragment (**right**). Changes in pH are transmitted through M2 to the virion interior, where they cause M1 to dissociate from RNPs. The increasing acidity also causes conformational changes in HA, which inserts into the endosomal membrane and mediates membrane fusion, expelling a complex of uncoated RNPs into the cytoplasm. The complex of RNPs interacts with the classical nuclear import pathway and dissociates in the nucleus (**bottom**). See text for details.

Influenza infections are caused by members of the orthomyxovirus family. The best-studied genus of the family is the influenza A viruses, the leading cause of influenza in humans and in a wide range of mammals and birds. Influenza B and C viruses also cause influenza in humans and in a more limited range of mammals, and distinct clinical or veterinary diseases are caused by the other genera of the family, the Thogoto, Quaranfil and infectious salmon anaemia viruses [1]. The genomes of orthomyxoviruses are composed of single-stranded, negative-sense RNA, which is divided into multiple segments (eight in the case of influenza A viruses). Each segment is encapsidated by viral proteins into a ribonucleoprotein complex (RNP; Figure 1). Unlike the majority of RNA viruses, for which the replication cycle is confined to the cytoplasm, in the orthomyxovirus family RNPs enter the nucleus of an infected cell to transcribe and replicate. As a result, a newly-synthesised RNP must be transported through a number of cellular compartments, as well as between cells, in order to complete a cycle of infection (Figure 2 and Figure 3). This requires the RNP to interact with multiple cellular transport systems, and a number of recently-published studies have significantly increased our understanding of the mechanisms that transport the RNP through different stages of its journey. In this review, we will follow the course of an influenza A virus RNP from its synthesis in the nucleus of an infected cell through to its entry into another cell’s nucleus, and describe the cellular mechanisms that the RNP recruits for transport along its way.

## 2. RNPs: The Basic Units of Orthomyxoviruses

The virions of orthomyxoviruses are involved only in a single stage of the replication cycle, the passage of RNPs from cell to cell, and are disassembled upon viral entry. The viral RNPs, by contrast, maintain their structural integrity from their synthesis in the nucleus of an infected cell through to their entry into the nucleus of a newly-infected cell [1]. RNPs are the minimal replicative units of orthomyxoviruses. When reconstituted in cells they are capable of transcription and replication in the absence of other viral proteins [2,3,4,5], and reconstitution of a full set of RNPs is sufficient to initiate an infection [6,7,8]. The virions themselves are pleomorphic, typically do not contain enough functional RNPs to produce infections particles without additional RNPs from co-infecting virions and, in a co-infection, can package RNPs derived from different strains (discussed in Section 4.4, below). Thus it is the RNPs, rather than the virions that temporarily contain them as they pass between cells, that provide the basic units of an orthomyxovirus.

Structurally, each RNP consists of a segment of single-stranded negative-sense RNA, with the terminal sequences of the RNA bound by a trimeric RNA-dependent RNA polymerase and the remaining sequence bound by multiple copies of nucleoprotein (NP; Figure 1a,d). Bound NP oligomerises into a double-helical, rod-shaped structure [9,10]; a basic groove on the NP molecules binds the phosphate backbone of RNA without sequence specificity, leaving the RNA bases exposed (Figure 1d) [11,12]. 

## 3. Orthomyxoviruses: The Nuclear Family

Having RNPs enter the nucleus to transcribe and replicate provides orthomyxoviruses with a problem, but also with a number of advantages. The problem is that of crossing the nuclear envelope. Passage across the nuclear envelope takes place through nuclear pore complexes (NPCs) and for structures larger than 20–30 kDa this is a tightly regulated process mediated by nuclear transport receptors, collectively referred to as karyopherins, that shuttle backwards and forwards through the NPC under the influence of a concentration gradient of Ran-GTP [13]. To gain entry to the nucleus, viral proteins must contain nuclear localisation signals (NLSs), which are bound in the cytoplasm by karyopherins known as importins and released when the importins have crossed into the nucleus and bind Ran-GTP. Crossing in the opposite direction requires a nuclear export signal (NES) that is bound by an exportin-Ran-GTP complex in the nucleus and released, along with Ran-GTP, in the cytoplasm. To exploit the nuclear compartment, orthomyxoviruses must interact with these import and export pathways. Functioning within the nuclear compartment has advantages for both transcription and replication. Replication within the nucleus allows viral RNA to be encapsidated into RNPs before it encounters the cytoplasmic RNA receptor retinoic acid inducible gene 1 (RIG-I) and triggers an antiviral response [18]. Transcription within the nucleus allows RNPs to associate with host RNA polymerase II (Pol II) [19,20,21,22], ensuring that they are well-positioned to ‘cap-snatch,’ cleaving short sequences including the 5´ cap structure from nascent host mRNAs and using these as primers for viral transcripts. Cap-snatching allows viral proteins to be translated and the resulting degradation of host transcripts, along with an increase in Pol II degradation [22,23,24,25], leads to host shutoff [26]. In addition, positioning viral transcription at the site of host transcription allows the virus to exploit host pathways of mRNA processing and export and to expand the coding capacity of the viral genome through splicing [27]. Splicing has been observed in transcripts from all orthomyxovirus genera [1]. In the case of influenza A virus spliced transcripts are generated from segment 7 (to produce the M2 and M42 proteins [28,29]) and segment 8 (to produce the nuclear export protein (NEP) and NS3 [30,31]).

## 4. From Synthesis to Virions

The assembly of a new RNP and its transport within an infected cell is illustrated in Figure 2 and described below.

### 4.1. Synthesis

Synthesis of a new RNP requires replication of the viral genome, as well as transcription of the genome and translation of the viral proteins that will encapsidate the genome. Transcription is performed by RNPs within the nucleus which copy their negative-sense viral RNA to produce positive-sense mRNA. Influenza A virus infection inhibits non-canonical translation in the nucleoli but not the low levels of non-canonical translation that can be detected elsewhere in the nucleoplasm, and it is therefore possible that some viral proteins are synthesised within the nucleus itself [32]. However, the majority of protein synthesis in infected cells appears to occur canonically within the cytoplasm [32] following mRNA export (reviewed in [27]). This leaves the problem that newly synthesised polymerase subunits and NP must be transported into the nucleus for the RNP to assemble.

To achieve this, the polymerase subunits and NP all contain NLSs (reviewed in [33]). The importins of different species differ slightly in their binding preferences, and consequently NLSs are among the sites that mutate as influenza A viruses adapt to new host species (reviewed in [34]). Interactions with import factors are used to chaperone RNP assembly and limit it to the nuclear compartment. The polymerase acidic (PA) and polymerase basic 1 (PB1) subunits are only efficiently imported into the nucleus after forming a dimer in the cytoplasm, whereas the polymerase basic 2 subunit is imported separately and may undergo a conformational change on importin binding that promotes its binding to the PB1-PA dimer [35,36,37,38,39,40]. Influenza viruses recruit cellular chaperones at various stages in their replication cycle [41,42,43,44], and interactions with importins have been proposed to chaperone PB1 and NP prior to RNP assembly [33,45,46].

Transcription and replication are two distinct modes of RNP function (reviewed in [47]). Both involve copying the negative-sense viral RNA (vRNA) to produce a positive-sense reverse-complement. A transcribing RNP produces a partial copy of the vRNA template, which is capped and polyadenylated. In contrast, a replicating RNP produces a full-length complementary RNA (cRNA), which must be copied again to produce a new copy of the vRNA. Newly-synthesised vRNA is encapsidated co-transcriptionally by a viral polymerase and free NP to form an RNP [10,48]. Early in infection transcribing RNPs dominate, with replication becoming more common as the infection progresses [25,49]. What causes an RNP to replicate its RNA rather than transcribe it is unclear, though a number of factors have been implicated [47]. Recent studies suggest that the two modes of activity differ in the source of the polymerase. Transcribing polymerases can function in *cis*, copying the RNA of their RNP. Replication, by contrast, requires a free polymerase, such as that assembled from newly-translated and imported viral proteins [50]. The cRNA-containing RNP appears to be a replicative intermediate and does not accumulate to high levels [51,52,53]. While cRNA-containing RNPs do not appear to be exported from the nucleus [54], vRNA-containing RNPs must be exported if the infection is to proceed.

### 4.2. Nuclear Export

As the infection advances other viral proteins also accumulate in the nucleus. The matrix protein M1 contains an NLS [55], and its import into the nucleus and SUMOylation allow it to bind the newly-synthesised RNP through interactions with NP and possibly also with viral RNA [56,57,58,59,60]. The nuclear export protein (NEP) is only 14 kDa in size and small enough to pass through the nuclear pore without binding to importins; it binds in turn to M1 [61,62,63,64]. NEP contains two NESs, both recognised by the exportin Crm1 [65,66,67]. M1 and NEP link the RNP to Crm1 though a ‘daisy chain’ of proteins (reviewed in [68]). Additional roles in mediating nuclear export have been suggested for NP and M1 [69,70], both of which have recently been shown to contain NESs recognised by Crm1 (in addition NP has been reported to have two Crm1-independent NESs) [71,72]. Crm1 is activated for nuclear export by binding to Ran-GTP, which is generated in the nucleus by the chromatin-bound Ran guanine exchange factor Rcc1. Prior to export RNPs are tethered to the same regions of chromatin as Rcc1, an association which presumably increases the efficiency of nuclear export [73].

The nuclear export of RNPs is a regulated process, with a number of separate mechanisms promoting export at late time points in infection. The progression of infection triggers the apoptotic pathway, promoting RNP export through the activation of caspase 3 [74,75], possibly because activated caspase 3 increases the diffusion limit of nuclear pores [76]. Accumulation of the viral surface protein haemagglutinin (HA) at the cell surface late in infection activates the mitogen-activated protein kinase (MAPK) signalling cascade [77], which in turn enhances RNP export [78]. Although this effect is presumably mediated by a kinase in the cascade, inhibition of the pathway did not detectably alter M1, NP or NEP phosphorylation *in vivo*, and the mechanism of action is currently unknown [78]. Conversely, although a phosphorylation site on NEP has been mapped to a short interhelical loop between the two NESs — an arrangement similar to other proteins in which phosphorylation regulates the exposure of an NES — the kinase that modifies this site has not yet been identified [79]. The best-understood mechanism regulating RNP export is the slow accumulation of NEP during an infection. NEP mRNA is generated by splicing at a weak 5´ splice site [80], resulting in a low rate of NEP synthesis compared to proteins generated from unspliced viral mRNAs. A recent study showed that NEP levels correlate with RNP export, and that the slow accumulation of NEP results in a ‘molecular timer’ that promotes RNP export only at late time points of infection (Figure 1e,f) [81].

The epithelial cells infected by influenza are polarised, and in order to spread to new cells RNPs assemble into virions at the apical plasma membrane [82]. Intriguingly, indirect immunofluorescence imaging of NP suggests that RNPs begin to move towards the apical surface even before nuclear export, and accumulate near the apical face of the inner nuclear membrane [83]. The mechanism for this apical polarisation of the nuclear compartment is unknown.

### 4.3. Trafficking to the Plasma Membrane

Nuclear export transports RNPs to the perinuclear cytoplasm, where they can be seen to accumulate by immunofluorescence (Figure 1e) [17,84,85,86,87]. This region contains the microtubule organising centre (MTOC) and the accumulation of RNPs here may be partly explained by Y-box Binding protein 1 (YB-1), which binds to RNPs in the nucleus and is able to interact with microtubules after nuclear export [88]. The cellular Human immunodeficiency virus Rev Binding protein (HRB) may assist in dissociating Crm1-Ran-GTP from the complex, and in facilitating onward trafficking of the RNP [89]. Near to the MTOC, RNPs are able to interact with recycling endosomes (REs) through Rab11, a GTPase which associates with REs in a GTP-dependent manner [17,87,90,91,92]. The viral polymerase, possibly through PB2, binds to the active (GTP-bound) form of Rab11, which in turn can bind to the RE and to various interacting partners that mediate vesicular transport to the apical plasma membrane [87,91,92]. Rab11 has two isoforms. To date most studies with influenza have concentrated on the role of Rab11A, though Rab11B has also been shown to be required for viral replication in a high-throughput screen [93].

By interacting with Rab11 on REs RNPs are able to use the vesicular transport system to move through the cytoplasm along the microtubule network (Figure 1e) [85,87,90,91]. Disruption of the microtubule network has been shown to reduce apical accumulation of RNPs [86,87], but it is not the only means by which RNPs are transported through the cytoplasm. Some RNPs appear to migrate slowly away from the perinuclear region by diffusion [87,91,94], whereas others make short-range movements along actin filaments [87,91]. At late time points in infection RNPs accumulate on large, Rab11-containing structures adjacent to the plasma membrane [17]. From there RNPs migrate to the apical plasma membrane, but Rab11 does not transfer with them, and nor is it incorporated into virions (Figure 1f) [17,95]. The RNPs have been released, presumably due to the hydrolysis of GTP converting Rab11 to its ‘inactive’ GDP-bound form.

In addition to transporting RNPs to the apical plasma membrane, the REs provide a platform for RNPs to come together and interact. Consistent with this, co-localisation of RNPs is significantly higher when they are bound to Rab11 [96]. As each RNP only contains a segment of the viral genome, the chances of a successful infection are greatly increased if the RNPs of different segments can associate with each other when they are packaged into virions. At the point of packaging (see Section 4.4, below), complexes of eight parallel and closely apposed RNPs can be observed (Figure 1b,c). Electron tomography has shown that these are composed of segments of distinctive lengths, with only a small number of the possible orderings of segments observed [16,97,98,99,100]. RNA packaging signals are used to bring specific segments together into a complex (reviewed in [101]), thus increasing the likelihood of all eight segments being packaged. Packaging signals are known to reside in the terminal regions of each segment, including both coding and non-coding sequences [101,102], and may also involve RNA across the entire length of a segment [99,100]. Recent *in vitro* studies have shown that RNA bases in the packaging signals interact directly to bring segments together [97,98,99]. These interactions appear to result in a ‘hierarchy of packaging,’ with certain segments, notably segments 1 and 7, having particular importance in co-ordinating interactions between RNPs [101,103,104,105,106,107,108,109]. It seems likely that binding to Rab11 facilitates these specific interactions between RNPs as it increases their local concentration, imposes a consistent orientation on them, and reduces their movement from free diffusion in three dimensions to lateral diffusion across two dimensions on a membrane [90].

Until they begin to associate on the surface of REs, RNPs appear to be essentially independent of each other [96,110]. The high error rate of the viral polymerase and the possibility of co-infection of a cell by more than one virus means that there is considerable diversity in the pool of newly-copied RNPs. At the REs different combinations from this pool can assemble into complexes of RNPs that comprise different reassortments of the viral genome. During natural co-infections between closely related strains this reassortment of the genome has been shown to occur with extremely high efficiency [111]. It allows influenza viruses to combine rapid genetic drift with the ability to restore genomes free of deleterious mutations, as in a co-infection between viruses with lesions in different segments reassortment allows the restoration of a genome without lesions [101,112,113].

Reassortment of genomes between distantly-related viruses within the same genus may be less efficient, partly because of incompatibilities between the viruses’ gene products [114] and also because of divergence between the packaging signals of avian and mammalian viruses [99]. When genomes from distantly-related viruses do reassort however, the genetic shift that results can greatly facilitate viral evolution, replacing epitopes to which the host has existing immunity and introducing host-adaptation and drug resistance traits. Genetic shift has played a major part in the evolution of most modern influenza pandemics [101,115,116].

### 4.4. Virion Assembly

As the infection progresses, the apical plasma membrane becomes enriched with viral proteins, which together co-ordinate the budding of virions around the complexes of RNPs. As reviewed in [117], the glycoproteins haemagglutinin (HA) and neuraminidase (NA) span the membrane and are concentrated in lipid raft microdomains. The membrane is also spanned by the ion channel M2, which accumulates on the boundaries of lipid rafts. On the cytoplasmic face of the membrane, the matrix protein M1 interacts with the cytoplasmic tails of HA, NA and M2 and with the membrane itself. Both M1 and M2 can interact with RNPs, and HA, NA, M1 and M2 can, when individually over-expressed, cause the budding of virus-like particles. How viral proteins interact during the formation of an actual virion is, however, still poorly understood. It is reasonable to assume that in an infection budding is promoted by the arrival of complexes of RNPs at the cell surface. Indeed, mutations in genome packaging signals that disrupt the formation of complexes of RNPs can reduce budding, though this effect appears to be at least partly cell-type dependent [101,104]. It has been suggested that RNPs may mediate budding through interactions with M1. The conformational change of M1 upon RNP binding may cause it to polymerise, driving capsid formation, or may reduce the ability of M1 to alter membrane curvature and so allow for the elongation of a budding event initiated by HA and NA. However, evidence to support these models is currently lacking [117]. In addition to viral factors, host factors required for budding include G-protein and kinase activity, as well as ATP, F1Fo-ATPase activity and actin filaments [118,119,120,121]. Rab11 has been shown to be required for budding, though this may be due to its role in transporting RNPs to the cell surface [84,92]. An interaction between M1 and RACK1, an adaptor protein involved in RE trafficking, is also required for viral budding [122]. 

Although the precise mechanism of virus budding is unknown, its effects can be clearly visualised by electron microscopy. The plasma membrane, densely packed with HA and NA, extrudes outwards from the cell. Inside, underneath a layer of M1, a complex of parallel RNPs occupies the distal tip of the nascent virion (Figure 1b). Electron tomography shows that, despite their different lengths, the RNPs are aligned at the distal end of the virion, and make close connections with each other within a ‘transition zone’ large enough to contain the terminal packaging signals [16,97,98,100]. Budding is completed by membrane abscission, mediated by the M2 protein [123]. In laboratory strains this often occurs more or less immediately after the packaging of RNPs, forming spherical virions around 100 nm in diameter. However, in clinical strains many virions often continue the budding process until they have formed extended filaments, which still contain only one complex of RNPs but can be many microns in length [16,124,125,126,127,128,129]. The production of filamentous virions is cell-type dependent [130] and has a particular requirement for actin and for the Rab11 family interacting protein 3 (FIP3), which interacts with cortical actin through Arf6 [84,130,131]. Despite their apparent abundance in clinical strains, filamentous viruses are rapidly selected against by passage in tissue culture and in embryonated chicken eggs, and the most widely-studied laboratory strains only produce spherical virions [132,133,134,135]. In part because of this, the biological function of filaments is obscure. 

Extensive studies have shown that influenza A virions do not typically package more than eight segments [16,97,98,100,101,136], with each segment typically present no more than once per virion [137,138,139,140,141]. Studies of low-multiplicity infections show that the vast majority of virions are not independently infectious and fail to express one or more viral genes on entering a cell [104,109]. Interestingly, this effect appears to vary between strains of virus—for example, the filamentous influenza A/Udorn/307/72 virus produces a higher proportion of fully-infectious virions than the spherical influenza A/Puerto Rico/8/1934 virus [109]. Failure to detect a particular segment could be due to the packaging of a defective RNP, or to inefficient packaging of RNPs. Both of these deficiencies have been shown to occur. As well as point mutations, segments of influenza are subject to large internal deletions, producing defective interfering (DI) RNAs [15]. Pairwise measurements of segment co-localisation suggest that virions containing segment 1 package the other seven segments of the genome very efficiently [141]. However, inferring the true packaging efficiency is complicated by the hierarchical nature of packaging—segment 1 has a particularly strong influence on the packaging of other segments, and the efficiency of packaging in any segment-1-deficient viruses is likely to be reduced [103,105,108]. Consistent with some degree of under-packaging, viruses containing only seven segments can be produced artificially and can be detected in natural infections [142,143]. Whether because of incomplete genome packaging or the packaging of defective RNPs, the majority of RNPs that are packaged into virions will subsequently require high-multiplicity infections in order to propagate [109].

Once the virion is released the RNPs have no direct influence on their journey. Within the virion the complex of RNPs may become somewhat disordered, with closely-packed bundles of eight parallel segments less apparent in virions [101,136]. Despite this, the RNPs appear to maintain their association with each other while the virion drifts away from the infected cell [96].

## 5. From Virions to the Nucleus

The entry of RNPs into newly-infected cells and their transport into the nucleus is illustrated in Figure 3 and described below.

### 5.1. Entry

Influenza virions attach to glycoproteins on the apical cell surface, an interaction brought about by viral HA binding to terminal sialic acids [1]. This leads to uptake of the virus either through clathrin-dependent receptor-mediated endocytosis, an AP-2-independent process that utilises the adaptor epsin 1, or through macropinocytosis [144,145,146,147,148]. The efficiency of uptake is increased by signalling events triggered when HA binds to receptor tyrosine kinases [149]. Filamentous virions are too large to fit into a clathrin-coated pit and must enter the cell through macropinocytosis [144]. Spherical virions can use either route, and the balance between routes of entry appears to be cell-type dependent [146,150]. The internalised virions are trafficked to an endosomal compartment; in the case of clathrin-mediated endocytosis by dynamin-dependent trafficking to a rapidly-maturing dynamic early endosome and in the case of macropinosomes via an unknown, dynamin-independent route [145,146,148,151]. As the endosome matures, the acidity of the endosomal lumen increases to around pH 5.2 [152]. This triggers a number of changes in the virion. In response to increasing acidity, M2 undergoes conformational changes that in turn alter the curvature of the viral membrane, fragmenting filamentous virions into spherical particles [144]. M2 also functions as an ion channel, allowing the virion interior to acidify [153]. As a result, the changing pH causes M1 to dissociate from the RNPs [154]. Finally, the acidic conditions of the endosomal lumen cause a dramatic conformational change in HA, exposing a fusion peptide. This inserts into the endosomal membrane, and HA then folds back on itself to bring the viral and endosomal membranes together, forcing the membranes to fuse and releasing the viral contents into the cytoplasm [155]. It appears that between a third and a half of virions fail to escape from the endosome before it fuses with a lysosome and are degraded [156,157]. For those that do manage to fuse in time, the uncoated RNPs are ejected into the cytoplasm of a newly-infected cell.

### 5.2. Nuclear Import

To complete the viral replication cycle, the complex of RNPs must enter the cell’s nucleus. Transport of RNPs from the endosome to the nuclear membrane appears to rely on diffusion, with neither microtubules, intermediate filaments, nor actin filaments required [94,156]. The trafficking is rapid and RNPs can be detected within the nucleus within the first ten to twenty minutes of infection [96,156]. The complex of RNPs maintains its association in the cytoplasm, only dissociating in the nucleus [96,158].

An RNP synthesised in a cell and exported from the nucleus is not imported back again, as its NLSs are masked by its coat of M1 [154,159,160] and possibly also by additional, M1-independent mechanisms [161]. By contrast, RNPs entering a cell from a virion are not coated with M1 and are competent for nuclear import. Nuclear import of uncoated RNPs is mediated by the classical nuclear import pathway, with the NP components of RNPs binding to alpha importins, which in turn bind to importin β to allow nuclear import [156,162]. NP contains at least two NLSs that are involved in importing newly-synthesised NP [33]. The same two NLSs recruit importins to the RNP, with the non-classical NLS1 at the N-terminus being more accessible within the structural context of the RNP [161,163,164,165,166]. Prior to entering the nucleus RNPs interact with NPCs, and may undergo several rounds of binding and release prior to nuclear import [94].

As discussed above (Section 4.1), the interaction of NP and the polymerase subunits with importins is a point of host-adaptation. The interaction of RNPs with importins also varies between hosts, though in a more complex fashion — for example, a mutation in PB2 associated with host adaptation affects the ability of the RNP to interact, through NP, with mammalian importins α1, α5 and α7 [167].

### 5.3. A New Cycle of Infection

In the nucleus, Ran-GTP binding to the importins displaces them from the RNP. It appears that this is the point at which the complex of RNPs dissociates, though what triggers this is unclear [96]. Now independent of each other again, the RNPs spread out into the nucleoplasm through diffusion [94] and begin to transcribe and then to replicate their genes. Another cycle of infection has begun.
